# Low Horizontal Force Production Capacity during Sprinting as a Potential Risk Factor of Hamstring Injury in Football

**DOI:** 10.3390/ijerph18157827

**Published:** 2021-07-23

**Authors:** Pascal Edouard, Johan Lahti, Ryu Nagahara, Pierre Samozino, Laurent Navarro, Kenny Guex, Jérémy Rossi, Matt Brughelli, Jurdan Mendiguchia, Jean-Benoît Morin

**Affiliations:** 1 UJM-Saint-Etienne, Laboratory Interuniversity of Human Movement Sciences, University Lyon, EA 7424, F-42023 Saint-Etienne, France; jeremy.rossi@univ-st-etienne.fr (J.R.); jean.benoit.morin@univ-st-etienne.fr (J.-B.M.); 2 Sports Medicine Unity, Department of Clinical and Exercise Physiology, Faculty of Medicine, University Hospital of Saint-Etienne, CEDEX 2, F-42055 Saint-Etienne, France; 3 LAMHESS, Université Côte d’Azur, F-06200 Nice, France; lahti.johan87@gmail.com; 4 Sports Research and Development Core, University of Tsukuba, Ibaraki 305-8574, Japan; nagahara.r@gmail.com; 5 Faculty of Sports and Budo Coaching Studies, National Institute of Fitness and Sports in Kanoya, Kagoshima 891-2393, Japan; 6 Laboratory Interuniversity of Human Movement Sciences, University Savoie Mont Blanc, EA 7424, F-73000 Chambéry, France; pierre.samozino@univ-smb.fr; 7 Mines Saint-Etienne, Centre CIS, Université de Lyon, Université Jean Monnet, INSERM, U 1059 Sainbiose, F-42023 Saint-Etienne, France; navarro@emse.fr; 8 School of Health Sciences (HESAV), HES-SO University of Applied Sciences and Arts Western Switzerland, 1011 Lausanne, Switzerland; kenny.guex@gmail.com; 9 Department of Sprints, Hurdles and Relays, Swiss Athletics, Haus des Sports, 3063 Ittigen, Switzerland; 10 Sports Performance Research Institute New Zealand, Auckland University of Technology, 1010 Auckland, New Zealand; matt.brughelli@aut.ac.nz; 11 Department of Physical Therapy, Zentrum Rehabilitation and Performance Center, 31002 Pamplona, Spain; jmendiguchia@zentrumsport.com

**Keywords:** hamstring strain, sports injury prevention, risk factors, sprinting, injury surveillance, soccer, prospective studies

## Abstract

Clear decreases in horizontal force production capacity during sprint acceleration have been reported after hamstring injuries (HI) in football players. We hypothesized that lower *F_H_*_0_ is associated with a higher HI occurrence in football players. We aimed to analyze the association between sprint running horizontal force production capacities at low (*F_H_*_0_) and high (*V*_0_) velocities, and HI occurrence in football. This prospective cohort study included 284 football players over one season. All players performed 30 m field sprints at the beginning and different times during the season. Sprint velocity data were used to compute sprint mechanical properties. Players’ injury data were prospectively collected during the entire season. Cox regression analyses were performed using new HI as the outcome, and horizontal force production capacity (*F_H_*_0_ and *V*_0_) was used at the start of the season (model 1) and at each measurement time point within the season (model 2) as explanatory variables, adjusted for individual players’ (model 2) age, geographical group of players, height, body mass, and previous HI, with cumulative hours of football practice as the time scale. A total of 47 new HI (20% of all injuries) were observed in 38 out of 284 players (13%). There were no associations between *F_H_*_0_ and/or *V*_0_ values at the start of the season and new HI occurrence during the season (model 1). During the season, a total of 801 measurements were performed, from one to six per player. Lower measured *F_H_*_0_ values were significantly associated with a higher risk of sustaining HI within the weeks following sprint measurement (HR = 2.67 (95% CI: 1.51 to 4.73), *p* < 0.001) (model 2). In conclusion, low horizontal force production capacities at low velocity during early sprint acceleration (*F_H_*_0_) may be considered as a potential additional factor associated with HI risk in a comprehensive, multifactorial, and individualized approach.

## 1. Introduction

Hamstring injuries are the most prevalent injuries in football (soccer), with an average of 22% of players sustaining at least one hamstring injury during a season [1]. The majority of hamstring injuries (~70%) occur during high-speed sprinting actions such as winning ball possession, passing a defending player, or gaining position to score a goal [2]. Consequently, it seems logical to expect sprinting to be a key parameter in football from both performance and hamstring injury prevention perspectives.

Sprint acceleration performance has been shown to be associated with the ability to produce and apply high levels of force in the horizontal direction over the entire acceleration [3]. This ability is well described by a macroscopic linear relationship between horizontal force and velocity obtained during sprint acceleration (i.e., F-v relationship) [4,5]. The F-v relationship is an integrative descriptor of an athlete’s mechanical output capabilities during maximum sprinting accelerations, representing the maximal force an athlete can produce in the horizontal direction during sprinting at different velocities [4,5]. The left side of the spectrum represents the force production capacity at low velocities and the right side represents the force production capacity at high velocities. Using the two extremes of the curve and spectrum (axes intercepts) allows to not depend on the choice of specific velocities to characterize these two force production capacities. Consequently, *F_H_*_0_ (which is the theoretical maximal force production at zero velocity) represents the force production capacity at extremely low velocity, and *V*_0_ (which is the theoretical maximal velocity until which horizontal force can be produced) represents the force production capacity at extremely high velocity (for illustration, see Figure 1 of Cross et al. [6]) [4].

This ability to produce horizontal force is mainly associated with muscular actions in the posterior chain, including the hamstrings, gluteals, and plantar flexors [7,8,9], which is one of the hypotheses explaining the high rate of hamstring injuries during acceleration and sprint running activities. In the hamstring injury prevention approach, knee flexor strength has been analyzed as a risk factor, screening test, and prevention measure [10,11,12,13]. However, prospective studies in football have shown that isolated, single-joint torque knee flexor evaluations do not accurately predict hamstring injury [10,11,12,13]. Such an approach does not reflect the complex action of these bi-articular muscles during sprinting [9,14,15]. This supports the importance of analyzing a complex phenomenon such as sprinting not only through an isolated part or sum of parts (one or more single muscles), but also through the actual specific behavior of the entire neuromuscular system of the lower extremity during sprinting (i.e., integrative macroscopic approach) [16,17,18,19]. This may eventually better reflect the overall ability of the athlete to develop horizontal force on the ground during the specific sprint running task, contrary to single-joint, isolated, and nonspecific evaluations.

In line with the close relationships between sprinting mechanics (especially the F-v profile) and hamstring function, a clear decrease in *F_H_*_0_, with no change in *V*_0_, has been reported at the time of return to football after hamstring injury [20,21]. This decrease in *F_H_*_0_ could be related to the impairment of hamstring muscles to efficiently manage force production during sprinting. *F_H_*_0_ may be considered as an indirect marker of the entire posterior chain force production in the high-force, low-velocity context characterizing early acceleration. This reported *F_H_*_0_ decrease [20,21] is consistent with the reduced hamstring muscle strength reported after hamstring injury [22,23]. However, these latter results on *F_H_*_0_ decrease [20,21] do not inform about the extent to which the lower *F_H_*_0_ measured after hamstring injury is a consequence of the injury, or was already present before the injury and was part of the injury risk factors. This supports the interest of the present study.

In this context, we hypothesize that the lower horizontal force capacity (*F_H_*_0_ and *V*_0_) can indirectly reveal an overall functional weakness of the posterior chain, including the hamstring muscles. This may also be a marker of a previous injury as previously reported by Mendiguchia et al. [20,21], or risk for a future injury as hypothesized in the present study, which is not addressed in previous research. In addition, since few studies have reported information on running-based measurements as hamstring injury risk factors [24] or regarding the main hamstring injury mechanisms [2], and given arguments related to the interest of horizontal force production during sprinting [20,21], there is great interest in improving the knowledge of such potential hamstring injury risk factors in football. We therefore aimed to analyze the association between horizontal force production capacities during sprinting (*F_H_*_0_ and *V*_0_) and hamstring injury occurrence in football players. We hypothesized that lower *F_H_*_0_ is associated with higher hamstring injury occurrence in football players.

## 2. Materials and Methods

### 2.1. Study Design

We conducted a one-season prospective cohort study on football players who were assessed for mechanical outputs during sprint accelerations (i.e., horizontal force production during sprinting) in field conditions at the start of the season as well as several times during the season. For these players, prospective exposure and injury data collection was done throughout the entire season. The study protocol was reviewed and approved by the Saint-Etienne University Hospital Ethics Committee (institutional review board: IORG0007394; IRBN322016/CHUSTE).

### 2.2. Population

At the start of the season, we contacted the head coaches of football groups to ask for participation in this study. We explained the objectives, procedure, and risks of the study, via oral communication and a paper information sheet, to the head coaches, assistant coaches, and medical staff members (one per football team).

The football players were then included according to the following inclusion criteria: (1) considered fit for competitive football activity by the team medical staff, (2) normally involved in training sessions at the start of the season, and (3) free of injury and able to perform the first sprint acceleration mechanical output measurement at the start of the season. The exclusion criteria were any medical problems contraindicated for football and sprint measurements at the start of the season and being a goalkeeper. Informed written consent was provided by each player before their participation in the study.

### 2.3. Sprint Acceleration Mechanical Output Measurements

Players were tested only if they were considered fit for competitive football activity by the team medical staff. Testing sessions were performed in the middle of a week (~72 h after a match, if any), outdoors, on the usual training surface (i.e., artificial turf), without rain, without wind in the direction of the sprint (i.e., headwind or tailwind), with usual football clothing and shoes, after a structured warmup (including approximately 5 min of jogging, 5 min of dynamic stretching (movement drill), and 2 × 10 m and 2 × 30 m sprints with increasing intensity, with small variations according to teams).

At each testing session, the instantaneous sprint velocity was measured during 2 maximal 30 m sprints from a standing start, separated by 3 to 5 min of rest. Running velocity was measured by means of a laser distance measurement system (LDM 301, JENOPTIK, Jena, Germany; sampling rate, 100 Hz) for the Japan group, and a radar system (Stalker ATS Pro II, Applied Concepts, Richardson, TX, USA; sampling rate, 46.87 Hz) for the France and Finland groups. Both devices were used in previous studies and provide accurate data for further fitting and computation [4,25]. The device was placed on a tripod 10 m behind the subjects at a height of 1 m corresponding approximately to the height of the subjects’ centers of mass.

Then, running speed time curves were fitted by a mono-exponential function, allowing step-averaged horizontal external anterior-posterior ground reaction force computations using a recently validated method [4,25]. From these data, the maximal power output associated with the antero-posterior component of the ground reaction force (*P_max_*) and the maximal theoretical force and velocity components of the F-v profile (*F_H_*_0_ and *V*_0_) were calculated [4,25]. *F_H_*_0_ and *V*_0_ values from the sprint trial with the highest *P_max_* were used for analysis. This method of calculation has been reported to have a low risk of bias in comparison to the gold standard (i.e., force platforms) (<5%) [4,25], and the intertrial reliability measured by the coefficient of variation was 3.5% and 0.6% for *F_H_*_0_ and *V*_0_ values, respectively [25].

### 2.4. Exposure and Injury Data Collection

At the beginning of the season, the history of previous hamstring injuries was collected for each included player by the medical teams using their medical files (prospective injury data collection from previous seasons) or a retrospective player’s medical interview if it was the first season for the player. Considering we only needed a binary response (i.e., a previous hamstring injury or not—as defined below), we assume that the recall bias was not major in our present study. In addition, anthropometrical parameters (age, height, and body mass) were assessed.

During the season, exposure in hours of football practice (i.e., training and competition) was collected weekly by coaches.

During the season, all new injuries were prospectively collected by the medical teams using a standardized report form. An injury was defined as: “A physical complaint or observable damage to body tissue produced by the transfer of energy experienced or sustained by an athlete during participation in training or competition, whatever its consequences with respect to impairments in connection with competition or training” [26]. Injury characteristics were recorded following the classification reported by Timpka et al. [26], including the severity (i.e., minor 1–7 days, moderate 8–28 days, serious >28 days). For the purpose of this study, the primary outcome was new hamstring injury, corresponding to an injury with a location recorded at the “posterior thigh” and type recorded as “strain/muscle rupture/tear” [26], whatever the consequence on football practice (i.e., we included time-loss and no-time-loss injuries). The diagnosis was made by a sports medicine physician generally based on the clinical examination, associated with imaging according to their decision. Sports medicine physicians were blinded for the results of sprint acceleration mechanical output measurements.

### 2.5. Sample Size Calculation

Based on the recommendation from Bujang et al. [27], we used the formula: *n* = 100 + 50*i* (*i:* number of independent variables) because we aimed to analyze two variables (*F_H_*_0_ and *V*_0_); we calculated an *a priori* sample size of 200 players.

### 2.6. Statistical Analysis

First, descriptive analyses were performed using frequency and percentages for categorical data, means and standard deviations (SD) for continuous variables, and incidence of hamstring injuries per 1000 h of football with a 95% confidence interval (95% CI), for the total population, as well as for the three groups (i.e., Finland, Japan, France) and between players with and without a history of hamstring injuries. The normality of all variables was tested using the Shapiro-Wilk normality test. Comparisons were performed on the baseline data (anthropometrical parameters, history of hamstring injuries, and baseline sprint acceleration mechanical outputs) between the three groups using ANOVA when data followed a normal distribution, and between players with and without history of hamstring injuries using Student’s t-test when data followed a normal distribution or Mann–Whitney U test if not.

Then, to analyze the association between horizontal force production capacities during sprinting (*F_H_*_0_ and *V*_0_) and hamstring injury occurrence, we used a time-to-event approach [28]; information after the occurrence of the outcome (i.e., hamstring injury) was censored. The time to first event was analyzed using cumulative hours of football practice (i.e., training and competition) as the time scale. A Cox proportional hazards regression (or Cox regression) model was used to analyze the association of *F_H_*_0_ and *V*_0_ with the occurrence of hamstring injury, adjusted to group, age, height, body mass, and history of previous hamstring injuries at the time of measurement (yes or no). A first adjusted Cox regression model (model 1) was conducted using the *F_H_*_0_ and *V*_0_ values at the start of the season and new hamstring injury occurring during the season (yes or no) as the outcome, with follow-up until the end of the season with no hamstring injury occurrence; the unit of analysis was the individual player. A second adjusted Cox regression model (model 2) was conducted using the *F_H_*_0_ and *V*_0_ values at each measurement session within the season and new hamstring injury occurring after the measurement session (yes or no) as the outcome, with follow-up when no hamstring injury occurrence until the next measurement, if any, or at the end of the season. The unit of analysis was the player-measurement and the Cox regression was also adjusted per individual player; analysis was adjusted to the individual player as some players completed more than one measurement [10]. The hazard ratio (HR) with a 95% CI was presented for each variable, and assumption that the HR was constant over time was tested.

The researchers who performed the analyses (PE and LN) were independent from football groups and did not conduct the measurements. Significance was accepted at *p* < 0.05. Analyses were performed using Excel (Office, Microsoft^®^, 2017, Redmond, DC, USA), JASP (JASP Team software, v0.11.1, University of Amsterdam, The Netherlands), and R (v3.6.3., © Copyright 2016 The Foundation for Statistical Computing, Vienna, Austria, (Comprehensive R Archive Network, http://www.R-project.org, accessed on 14 April 2020).

## 3. Results

### 3.1. Population and Sprint Acceleration Mechanical Output Measurements

**Figure 1:**
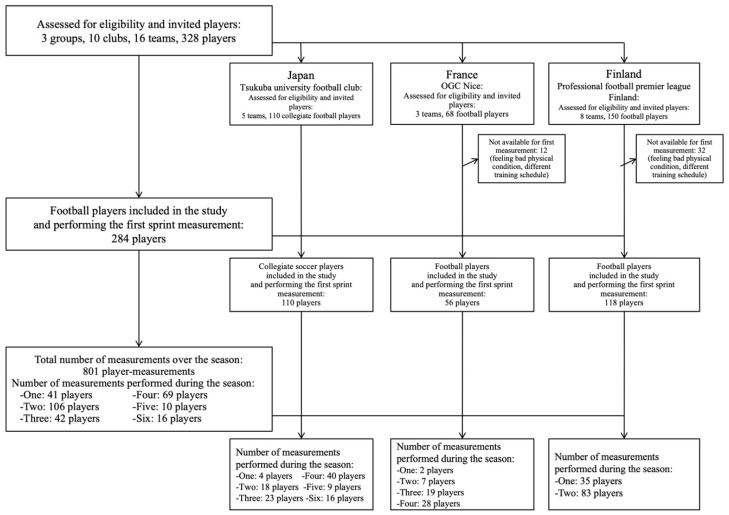
Flow chart showing the recruitment, number of included players and number of sprint acceleration.

We contacted the head coaches of three football groups: the Tsukuba university football club in Japan (including five football teams; Japan group) at the start of the 2014–2015 season; OGC Nice in France (including three football teams (second team, U19 and U17); France group) at the start of the 2017–2018 season; and eight teams of the professional football premier league “Veikkausliiga” in Finland (Finland group) at the start of the 2019 season. From the 328 eligible players (110 from Japan, 68 from France, 150 from Finland), 284 players were included in this study. The recruitment flow chart is presented in Figure 1, and the characteristics of the included players are presented in Table 1.

**Figure 2:**
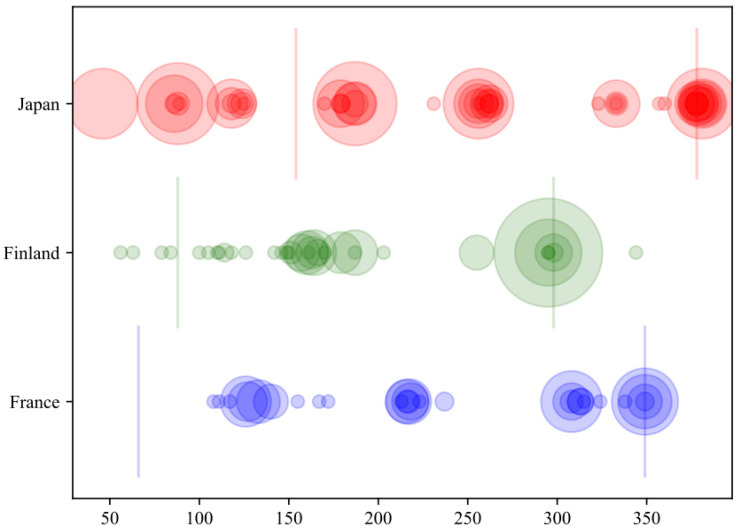
The timing of the sprint acceleration mechanical output measurements during the season. The x-axis represents the time (in days) from the start of the season (t = 0), the y-axis represents the groups (Finland = green, Japan = red, France = blue), the color of the circle represents the groups (Finland = green, Japan = red, France = blue), the size of the circle represents the number of measurements at the measurement session. The first vertical line represents the end of the pre-season and the second vertical line the end of the season, with color according to the group.

Among the 284 players, a total of 801 player-measurements were performed: 41 players performed one measurement (14%), 106 performed two measurements (37%), 42 performed three measurements (15%), 69 performed four measurements (24%), 10 performed five measurements (4%), and 16 performed six measurements (6%). The timing of the sprint acceleration mechanical output measurements during the season is presented in Figure 2.

### 3.2. Hamstring Injuries

A total of 47 new hamstring injuries occurred in 38 players (13%) (Table 1). An amount of 41 hamstring injuries (87%) lead to time loss from football, with a mean time absence of 18 (SD = 11) days; the severity was reported as minor for 8, moderate for 28 and serious for 5 hamstring injuries. No hamstring injury occurred during sprint acceleration mechanical output tests. The mean time between the measurement at the start of the season and the first hamstring injury occurrence was 166 (SD = 158) hours of football practice (model 1), and the mean time between a (subsequent) measurement and the first hamstring injury occurrence was 73 (SD = 48) hours of football practice (model 2).

### 3.3. Associations between Sprint Horizontal Force Production Capacities and Hamstring Injuries

When considering baseline data only (model 1), the adjusted Cox regression showed no association of *F_H_*_0_ or *V*_0_ with the occurrence of new hamstring injury (Table 2). When considering the *F_H_*_0_ and *V*_0_ values at each measurement session (model 2), the adjusted Cox regression showed that lower *F_H_*_0_ was significantly associated with higher occurrence of new hamstring injury (HR = 2.67 (95% CI: 1.51 to 4.73), *p* < 0.001), while *V*_0_ was not (Table 2).

## 4. Discussion

The main findings of the present study were that (1) horizontal force production capacity at low (*F_H_*_0_) and high (*V*_0_) velocities measured at the start of the season was not associated with a new hamstring injury occurring during the season, and (2) lower maximal horizontal force production capacity at low velocities (*F_H_*_0_) measured at more regular intervals of time over the season was significantly associated with a higher rate of new hamstring injury occurring within the weeks following the sprint acceleration mechanical output testing, while *V*_0_ was not. Every 1 N.kg decrease of *F_H_*_0_ was associated with 2.67 times higher risk of sustaining a new hamstring injury, within a mean timing of 73 h of football practice (corresponding at ~7 weeks with 10 h of football practice per week).

### 4.1. Strengths and Limitations

The main strengths were the numbers of included and injured players leading to a sufficient number of cases for analyses [27,29], the representativity of the population (football players, high levels, different countries and continents), the collection of sprint acceleration mechanical output data at different times of a football season (which is only rarely published) [30], the time-to-event analytical approach [28] and adjustment of analyses to important hamstring injury risk factors (i.e., football exposure, age, history of previous hamstring injuries) [24,31,32] and to the confounding factors (i.e., geographic location and individual player). In addition, sprint acceleration mechanical outputs were easy to assess in a sports practice context (performed on training field with few materials, did not require a significant change in players’ training), and were well accepted by players, coaches, and technical and medical staffs.

Regarding the limitations, heterogeneity of the population (different countries, playing levels, age groups, and training habits) might have had an influence on the results, therefore analyses were adjusted to the group and age of players. The number of measurements was different and measurements were performed at different times within the season depending on the teams and players, which limited the analyses of the changes in sprint acceleration mechanical outputs within the season [30] and over time in relation to new hamstring injury. However, we think that these limitations are outweighed by the fact that these measurements were performed on a large overall cohort and real-life scenario of high-level football practice. The results differed between models 1 and 2, explained by the fact that input data were different, which supports the interest of regular measurements within a season. Isolated hamstring muscle strength was not available, although it is reported inconsistently as an intrinsic risk factor [10,11,24,33,34,35]. The hamstring injury diagnosis was performed by different sports medicine physicians, according to teams, with potential different skills and diagnosis habits, however it was based on the same injury definition and classification. Not all hamstring injuries were confirmed by MRI. The sprint acceleration mechanical outputs were analyzed during linear sprinting, while hamstring injuries might have occurred during another movement other than sprinting (e.g., slide tackling (overstretch), cutting (change of direction), and kicking) [2], and the exact mechanisms of hamstring injuries were not available. Previous hamstring injuries were retrospectively collected, exposing the recall bias. The time from previous hamstring injury was not assessed, and this can change the impact on the horizontal force production and the occurrence of a new hamstring injury. This simple measurement is not an assessment of specific muscles that contribute to acceleration, nor does it take sprint running technique into consideration [36,37,38], and it does not take into account all the other potential injury risk factors [18,24,31,32]. This simple measurement of sprinting mechanical outputs is thus proposed as a complementary, sprint-specific component among other potential injury risk factors [24,31,32]. Finally, no analysis of specificity, sensitivity, or area under the curves for potential predictor variables was performed, as the study aim was to analyze the association between sprint mechanical outputs and hamstring injury, and the variability of *F_H_*_0_ and *V*_0_ values across the sporting populations and according to the levels [4,25,30] led to the risk of irrelevant and inappropriate cut-off values in practice.

### 4.2. Lower Maximal Horizontal Force Production as a Risk Factor for Hamstring Injury

After data collection regarding hamstring injuries [20,21], the present study adds new insights into using “pre-injury” data from sprint acceleration mechanical outputs. Our results show the association between new hamstring injuries and lower limb muscle function with a more integrative and performance-oriented approach than previously proposed [10,11,34,35,39]. This is also an approach to analyze the main injury mechanism of hamstring injury, i.e., high-speed sprinting [2].

As hip extensors and knee flexors, hamstring muscles are associated with the horizontal component of the ground reaction force during sprint running [8,9,40]. Any impairment of hamstring muscles can alter posterior chain power production and, in turn, result in a lower maximal horizontal force component [9,14,15]. We therefore suggest that the lower limb posterior chain function, including the hamstrings, gluteals, and plantar flexors, within the specific sprint running movement, may be indirectly assessed by measuring sprint acceleration mechanical outputs and notably *F_H_*_0_.

The impairment of hamstring muscles and its functional consequences may increase the risk of a new hamstring injury. Such an impairment can be considered revealing of an overall weakness of the muscles and posterior chain force production, which are not able to sustain the load required during sprint acceleration. It may also be considered as a marker of pre-injury without symptoms, which can progress to a symptomatic injury under increased loads. Thus, the measurement of the sprint acceleration mechanical output, and especially the *F_H_*_0_ component, can therefore be a promising, affordable, time-effective, and ecological screening tool for assessing the risk of hamstring or lower limb posterior chain injuries. Further studies should replicate this analysis to confirm the present results and analyze potential thresholds of *F_H_*_0_ values and predictive ability of this measurement to detect a higher risk of hamstring injuries. In addition, deeper analyses should be made in relation to the injury mechanism: is this association specific to sprinting hamstring injuries? Have *F_H_*_0_ and *V*_0_ a similar association with hamstring injury when injury occurs in the first steps, at or near maximal velocity, or during curve sprint?

### 4.3. The Multifactorial Nature of Hamstring Injury

We adjusted the analyses to parameters that have been reported in the literature to be associated with the hamstring injury risk [18,24,31,32]. This is important because these factors can influence the studied variables (*F_H_*_0_ and *V*_0_) in addition to the outcome (hamstring injury). This could be the case for the history of previous hamstring injuries, which is reported to be associated with a higher risk of new hamstring injury [24,31,32] and also with a decreased horizontal force production [20,21].

Such an approach, including adjustments of the analyses, is also consistent with the multifactorial nature of injury [41,42]. Hamstring injury is multifactorial as reported in the literature [24,31,32]; its prevention should be conducted accordingly [43]. This means that, in addition to considering several aspects related to the physical and biological characteristics (and not only focused on isolated factors such as strength, load, or flexibility) [18,44,45], psychological and sociological aspects should also be considered [43,46]. The context of hamstring injury should thus be included in the preventive approach [47], including for example, the psychological pressure (chocking) in team sports [48].

We suggest that hamstring injury management (prevention and rehabilitation) should be approached in a multifactorial way by keeping the main injury mechanism (sprint running) at the center of a comprehensive whole [17,18]. The latter is consistent with the fact that (i) field performance measurements, including sprint measurements, have been suggested as useful by experts for the return-to-sport decision [49], (ii) experienced field sports practitioners involved in professional football teams reported sprinting and high-speed running focused exercises as most effective (perceived) to prevent muscle injuries [50], (iii) running-based factors appear as potential hamstring injury risk factors [24], and (iv) the required running velocity in athletic disciplines has been reported to play a role in the hamstring injury risk [51].

### 4.4. Relevance of Regular Monitoring of Sprint Acceleration Mechanical Outputs over the Season

Given the fact that analyses using the measurement performed at the beginning of the season were not significant, while using all measurements throughout the season (e.g., measurements right before the injury) revealed a significant association with new HMI; we suggest that individual sprint acceleration mechanical outputs should be assessed regularly during the season. This is in agreement with Green et al. [24] suggesting that using data from a single occasion of baseline assessment may not be valid to prospectively evaluate associations with subsequent hamstring injuries occurring sometimes several months later. Sprint acceleration mechanical outputs may change with changes in physical status and training regimen throughout the season [30] and as a result of hamstring injuries [20,21]. Thus, regular measurements throughout the season are likely more valuable to better inform the player on injury risk. This will also be of interest to analyze the normal variability of weekly or monthly sprint acceleration mechanical outputs and their relation to hamstring injury risk, for example, promising in situ approaches recently presented by Morin et al. [52].

## 5. Conclusions

This prospective cohort study provides a new step in hamstring injury management by reporting a potential new hamstring injury risk factor and showing an interest of analyzing sprint acceleration mechanical outputs. This supports the interest of including sprinting in the hamstring injury risk reduction process as a piece of the puzzle to reach a solution [53]. Sprint acceleration mechanical outputs have been reported to be useful to orient training [5] and to return to sprinting after hamstring injuries [18,20]. Our results suggest that it may also help to inform players about the risk of hamstring injuries. Thus, this macroscopic, integrative, and ecological approach appears promising for both sprint performance improvement and hamstring injury management.

## Figures and Tables

**Table 1:** Baseline data including anthropometrical, history of hamstring injury, and sprint acceleration mechanical output data at the start of the season, in addition to new hamstring injuries (i.e., primary outcome), for the total population and according to the three groups (i.e., Finland, Japan and France) or to the history of hamstring injury.

	Total	Per Groups			Per History of Hamstring Injury	
		Japan	France	Finland	No Previous Hamstring Injury	History of Previous Hamstring Injury
Number (n (%))	284 (100)	110 (39)	56 (20)	118 (42)	224 (79)	60 (21)
Anthropometrical parameters (mean (SD))						
Age (years)	21.4 (4.3)	20.0 (1.0) ^c^**	18.2 (1.8)	24.3 (5.3) ^a,b^***	21.3 (4.1)	22.1 (5.2)
Height (cm)	176.7 (7.2)	172.7 (5.2) ^c^***	177.0 (6.7)	180.4 (7.0) ^a^***^,b^**	176.7 (7.2)	177.0 (7.0)
Mass (kg)	71.3 (8.4)	66.9 (5.3)	68.1 (8.1)	77.0 (7.6) ^a,b^***	71.4 (8.5)	71.2 (8.3)
History of hamstring muscle injury						
Number of players (%)	60 (21)	16 (15)	14 (25)	30 (25)	0 (0)	60 (100)
Sprint acceleration mechanical outputs (mean (SD))						
*P_max_* (W·kg^−1^)	16.8 (1.6)	16.3 (1.4) ^c^***	17.3 (1.8)	16.9 (1.6) ^a^**	16.7 (1.6)	17.1 (1.7)
*F_H_*_0_ (N·kg^−1^)	7.5 (0.6)	7.4 (0.6)	7.5 (0.6)	7.5 (0.6)	7.5 (0.6)	7.5 (0.6)
*V*_0_ (m·s^−1^)	9.0 (0.5)	8.8 (0.5) ^c^***	9.3 (0.6)	9.1 (0.4) ^a^***^,b^**	9.0 (0.5) ^d*^	9.1 (0.5)
*F-v profile*	−0.83 (0.08)	−0.84 (0.09)^c^*	−0.81 (0.09)	−0.83 (0.07)	−0.84 (0.09)	−0.82 (0.08)
New hamstring injury						
Number of players with new hamstring injury (n (%))	38 (13)	6 (6)	12 (21)	20 (17)	16 (7)	22 (37)
Number of new hamstring injury (n)	47	8	16	23	19	19
Incidence of new hamstring injury (per 1000 h of football (95% CI))	0.4 (0.3 to 0.5)	0.1 (0.0 to 0.2)	0.8 (0.5 to 1.2)	0.6 (0.4 to 0.9)	0.2 (0.1 to 0.3)	0.8 (0.4 to 1.2)

^a^ Finland differed from Japan and from France; ^b^ Finland differed from France; ^c^ Japan differed from France; ^d^ “No previous hamstring injury differed” from “History of previous hamstring injury”; * *p* < 0.05; ** *p* < 0.01; *** *p* < 0.001. 95% CI: 95% confidence interval. F-v profile corresponds to the slope of the F-v linear relationships. Among the 284 included players, a total of 241 injuries (95% leading to time loss in football) were reported during the season in 159 players (56%). The main injury diagnosis was ankle sprain (*n* = 52, 22%), followed by hamstring injury (*n* = 47; 20%); other injuries were lower limb non-muscle injuries except ankle sprain (26%), lower limb muscle injuries except hamstring injury (22%), and other injuries (10%). The incidence of hamstring injuries per 1000 h of football varied according the three groups (lower for Japan group) and between players with and without history of hamstring injury (higher in players with history of previous hamstring injury).

**Table 2:** Hazard ratio (HR) with 95% confidence interval (95% CI) of the association between *F_H_*_0_ and *V*_0_; and (1) new hamstring injury during the season based on *F_H_*_0_ and *V*_0_ values at the start of the season for the 284 players and adjusted for group, age, height, body mass, and history of hamstring injury (model 1); and (2) new hamstring injury after the sprint acceleration measurement based on the *F_H_*_0_ and *V*_0_ values of each measurement of the season for 801 player-measurements and adjusted for individual player, group, age, height, body mass, and history of hamstring injury (model 2), according to the time scale of cumulative hours of football.

	Model 1 (*n* = 284 Players)			Model 2 (*n* = 801 Player Measurements)		
Explanatory Variables	HR	(95% CI)	*p*-Value	HR	(95% CI)	*p*-Value
*F_H_*_0_ (N·kg^−1^)	1.27	(0.70 to 2.33)	0.43	2.67	(1.51 to 4.73)	<0.001
*V*_0_ (m·s^−1^)	1.31	(0.66 to 2.60)	0.44	1.49	(0.70 to 3.18)	0.30
Concordance (Harrell’s c-index)	0.817	(0.811 to 0.823)		0.937	(0.931 to 0.943)	

## Data Availability

Data are available upon reasonable request. Please contact the corresponding author.
